# Research Progress of Cuproptosis in Orthopaedics: Opportunities and Challenges

**DOI:** 10.1111/jcmm.71175

**Published:** 2026-05-10

**Authors:** Chaoshuai Wang, Pengfei An

**Affiliations:** ^1^ Shandong University of Traditional Chinese Medicine Jinan City Shandong Province China; ^2^ Weinan Central Hospital Weinan Shaanxi Province China

**Keywords:** arthritis, bone tumours, copper chelators, cuproptosis, osteoporosis

## Abstract

Cuproptosis, a recently identified and copper‐dependent form of regulated cell death, has emerged as a potential therapeutic target for a variety of diseases. This review synthesizes current evidence from PubMed to elucidate the mechanisms of cuproptosis and its implications in common orthopaedic disorders, including arthritis, bone tumours and osteoporosis. We systematically explore the dual role of copper homeostasis—highlighting how its dysregulation contributes to disease pathogenesis and how its targeted modulation may offer novel therapeutic strategies. Finally, we discuss the associated challenges and future directions for translating cuproptosis research into clinical orthopaedics practice.

## 1 Introduction

**FIGURE 1:**
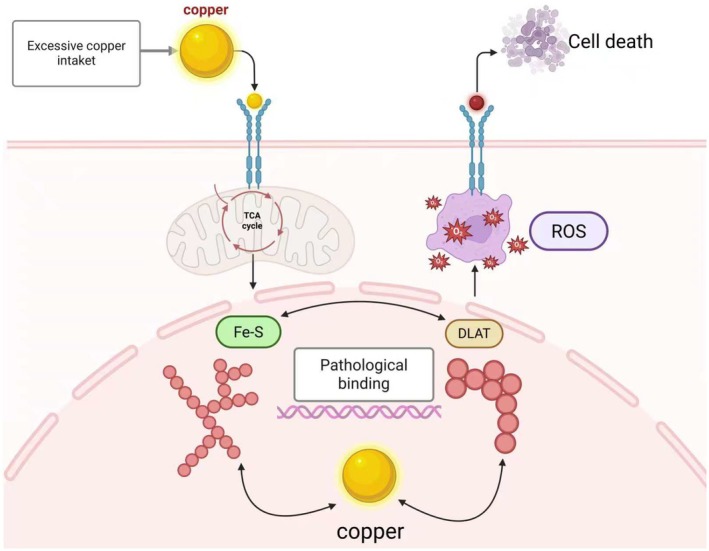
Diagram of the mechanism by which cuproptosis induces cell death. Excessive copper ions (Cu^2+^) infiltrate the mitochondrial matrix and disrupt the tricarboxylic acid (TCA) cycle. This leads to the aggregation of lipoylated proteins (e.g., DLAT) and the destabilization of iron–sulphur (Fe–S) cluster proteins, triggering proteotoxic stress and a surge in reactive oxygen species (ROS) production, ultimately culminating in cell death.

Orthopaedic disorders represent a major clinical and societal burden worldwide. Among them, arthritis and osteoporosis (OP) are highly prevalent in ageing populations. The chronic pain caused by arthritis and fragility fractures resulting from OP substantially impair patients' quality of life and may even lead to life‐threatening complications. Meanwhile, bone tumours—such as osteosarcoma (OS)—are highly malignant neoplasms with a peak incidence in adolescents and young adults, significantly reducing survival rates in this demographic and highlighting an urgent need for more effective treatments. In recent years, research on regulated cell death has expanded beyond traditional apoptosis. In 2012, Dixon et al. [1] first described ferroptosis, an iron‐dependent form of non‐apoptotic cell death. A decade later, Peter Tsvetkov and colleagues identified ‘cuproptosis’ [2] as a novel copper‐triggered cell death pathway (Figure 1). These discoveries underscore the diversity of metal‐dependent cell death mechanisms beyond ferroptosis. Cuproptosis is particularly unique due to its reliance on mitochondrial metabolism and its distinct mechanism of non‐apoptotic programmed cell death.

Copper (Cu), an essential trace element in humans primarily obtained from dietary sources, plays a critical role in maintaining physiological and pathological homeostasis [3]. Under normal conditions, systemic copper levels are tightly controlled by homeostatic regulatory mechanisms. However, disruption of intracellular copper homeostasis—leading to excessive copper accumulation—enables surplus copper ions to impair mitochondrial function, specifically within the tricarboxylic acid (TCA) cycle. Aberrant copper binding promotes abnormal aggregation of lipoylated DLAT, a component of the pyruvate dehydrogenase complex, and destabilizes iron–sulphur (Fe–S) cluster proteins in the TCA cycle [4]. These alterations induce proteotoxic stress, ultimately leading to a unique form of cell death termed cuproptosis [2]. Notably, copper overload resulting from homeostatic imbalance is closely associated with the pathogenesis of various diseases, including malignancies and neurological disorders [5, 6].

Given that cuproptosis is predominantly mitochondrial‐dependent, a deeper understanding of its mitochondrial mechanisms is essential for developing mitigation strategies. Mitochondrial enzymes and respiratory processes are central to the cuproptosis pathway [7]. Supporting this, Wu et al. demonstrated that variations in intracellular copper levels cause significant mitochondrial morphological changes [8]. Copper deficiency diminishes the activity of mitochondrial respiratory chain complexes [9] while copper overload triggers mitochondrial dysfunction and apoptosis [10].

## 2 The Potential Linkages of Copper to Orthopaedic Disorders

### 2.1 Copper in Osteoporosis (OP)

#### 2.1.1 Copper Homeostasis and Osteoporosis

OP predominantly affects elderly populations [11], significantly impairing quality of life in later years and imposing substantial economic burdens on families and society. Approximately 66% of total body copper is localized in bone and muscle tissues [12], highlighting its intrinsic importance to skeletal health and function. Osteoblasts and osteoclasts—two critical cell types in bone tissue—play essential roles in bone growth, repair, and remodelling. The structural and functional integrity of bone relies on a dynamic equilibrium between osteoblast‐mediated bone formation and osteoclast‐mediated bone resorption [13].

Copper plays a crucial role in regulating the activity of both cell types. It serves as an essential cofactor for lysyl oxidase (LOX) [14, 15], which catalyses collagen cross‐linking and enhances the mechanical strength of the bone matrix. Copper deficiency impairs LOX activity, leading to disorganized collagen fibres, increased bone brittleness, and consequently, elevated OP risk. Furthermore, copper is a key component of SOD, a potent antioxidant enzyme [16] that scavenges superoxide radicals generated during bone metabolism, thereby mitigating oxidative stress and preventing bone cell apoptosis and dysfunction.

Conversely, copper deficiency also exerts detrimental effects on skeletal health. As an essential cofactor for SOD, copper insufficiency compromises the antioxidant capacity of bone cells, leading to excessive accumulation of reactive oxygen species (ROS). This not only suppresses osteoblast proliferation but also promotes osteoclast activation via the NF‐κB signalling pathway. Furthermore, copper deficiency downregulates the expression of bone morphogenetic protein 2 (BMP2), a critical factor for osteoblast differentiation, thereby further inhibiting bone formation [13, 17]. Clinical studies have demonstrated that serum copper levels are significantly lower in elderly patients with OP compared to healthy controls, and copper supplementation (5–10 mg/day) has been shown to improve bone mineral density in patients with mild OP [18, 19].

When copper homeostasis is disrupted—particularly through excessive mitochondrial copper accumulation that overwhelms cellular regulatory capacity—copper transitions from a vital element to a cytotoxic trigger, inducing cell death via cuproptosis. Additionally, copper modulates osteoclast activity by promoting their differentiation while simultaneously suppressing bone‐resorptive function [20].

In summary, copper acts as a double‐edged sword in bone metabolism: deficiency impairs bone toughness and formation, increasing the risk of OP and fractures, whereas excess copper directly induces osteocyte toxicity via cuproptosis and disrupts bone metabolic homeostasis, ultimately compromising skeletal strength [21]. These findings underscore a significant association between copper dysregulation and OP pathogenesis.

#### 2.1.2 Cuproptosis in Osteoporosis (OP)

Copper homeostasis plays a fundamental role in bone metabolism, particularly in osteoblast function. Under physiological conditions, copper ions serve as essential cofactors that catalyse collagen and elastin cross‐linking, thereby promoting bone mineralization and enhancing mechanical strength [14, 15]. However, dysregulated copper homeostasis—specifically, excessive accumulation leading to cuproptosis—disrupts the normal activity of both osteoblasts and osteoclasts.

The detrimental effects of cuproptosis on osteoblasts are primarily mediated through two interconnected mechanisms. First, excess copper acts synergistically with oxidative stress [22]. As a highly redox‐active metal, elevated copper levels promote ROS generation via the Fenton reaction [23], resulting in oxidative damage that impairs osteoblast proliferation and differentiation, thereby suppressing bone formation [24]. Second, osteoblasts—being highly dependent on mitochondrial aerobic respiration for energy‐intensive processes such as collagen synthesis and matrix mineralization—are particularly vulnerable to cuproptosis, which directly targets lipoylated proteins within the mitochondrial respiratory chain [25].

At the molecular level, cuproptosis is initiated by a pathological rise in intracellular free copper ions (Cu^2+^), resulting from hereditary defects, environmental exposure, or inflammatory stimuli. These ions accumulate within mitochondria, where they disrupt the tricarboxylic acid (TCA) cycle, inducing oligomerization of lipoylated proteins and degradation of iron–sulphur cluster proteins. This cascade triggers severe proteotoxic stress, culminating in the rapid lytic death of osteoblasts and impaired skeletal remodelling [26].

**FIGURE 2:**
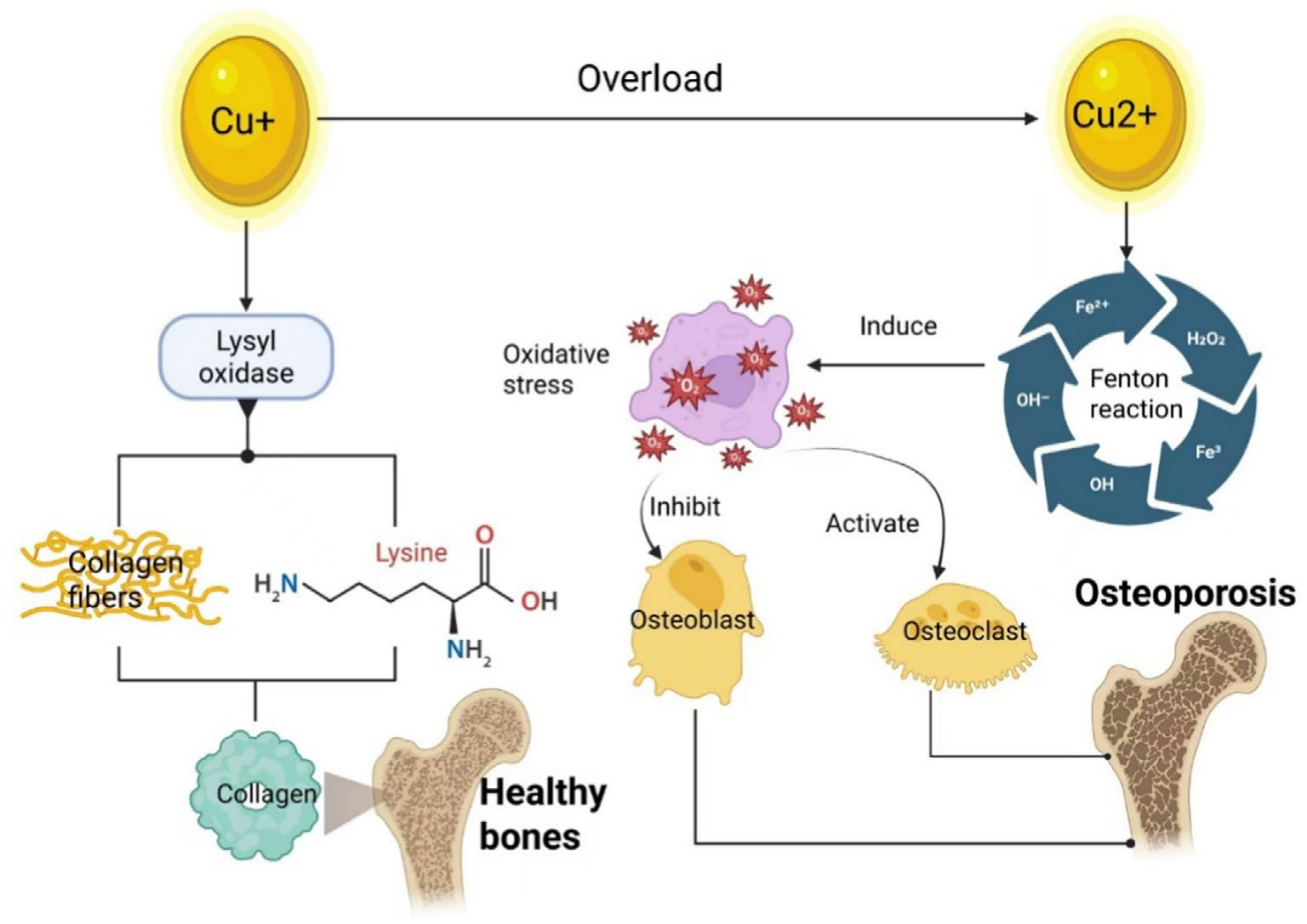
Mechanism diagram of copper and osteoporosis. (Left) Physiological copper ions act as cofactors for lysyl oxidase (LOX), facilitating the cross‐linking of specific amino acids (e.g., lysine) in collagen and elastin fibres. This process enhances collagen strength and stability in bone, maintaining skeletal integrity. (Right) Pathological copper overload induces cuproptosis and Fenton reaction‐driven oxidative stress within the bone microenvironment. This stress suppresses osteoblast activity and activates osteoclasts, ultimately leading to bone loss and osteoporosis.

Supporting these mechanistic insights, experimental studies have confirmed the osteotoxic effects of copper overload. Medeiros et al. demonstrated that excessive copper ions exert direct toxicity on bone and cartilage tissues [27], while Zhang et al. reported that elevated serum copper levels correlate with reduced bone mineral density in murine models [28]. Collectively, these findings indicate that cuproptosis contributes to OP pathogenesis by depleting functional osteoblasts and disrupting bone metabolic balance (Figure 2).

### 2.2 Copper in Osteoarthropathy

#### 2.2.1 Copper Homeostasis in Osteoarthropathy

Joints are complex structures composed of bone, cartilage, and synovium. With advancing age, the cellular and extracellular components of joints undergo progressive degeneration and cell death, gradually disrupting joint homeostasis. This process frequently leads to chronic joint pain and significantly impairs the quality of life in elderly populations. Epidemiological studies indicate that approximately one‐third of elderly individuals are affected by joint diseases [29], with osteoarthritis (OA) and rheumatoid arthritis (RA) representing the most prevalent forms [30].

In OA, pathological changes are primarily characterized by degenerative damage to articular cartilage. Interestingly, articular cartilage contains relatively high concentrations of copper [31]. Traditionally considered irreversible, advanced cartilage degeneration has been mainly managed through joint replacement surgery. However, recent advances in cartilage regeneration research have revealed that nanotechnology‐based approaches can promote articular cartilage reconstruction [32]. Although copper is not a structural component of joints, it plays indispensable roles in maintaining cartilage health. First, copper acts as an essential cofactor for LOX [14], which catalyses the oxidative deamination of specific lysine residues in collagen and elastin molecules. The resulting aldehyde groups spontaneously form stable covalent cross‐links between collagen and elastic fibres [33]. This cross‐linking is particularly crucial for articular cartilage, whose extracellular matrix consists predominantly of a dense type II collagen network that provides tensile strength and structural integrity [33]. Copper‐dependent LOX activity is therefore vital for maintaining the mechanical stability and resilience of the collagen architecture [34]. Copper deficiency or impaired LOX function leads to disorganized collagen fibrils and reduced mechanical strength, rendering cartilage susceptible to damage under load‐bearing stress [33, 35].

Second, copper is an integral component of SOD [36], a key antioxidant enzyme present in cytoplasmic and extracellular compartments. SOD catalyses the conversion of superoxide anions to oxygen and hydrogen peroxide, with the latter being degraded by catalase and other enzymes [37]. Under both physiological and pathological conditions such as OA, articular cartilage generates ROS. Excessive ROS damage chondrocytes, collagen, and proteoglycans, thereby accelerating cartilage degeneration [38]. SOD constitutes the primary defence mechanism against such oxidative stress [39], protecting cartilage components from oxidative damage.

The relationship between copper and RA also demonstrates a dual nature, reflecting its complex role in redox balance. Elevated oxidative stress is a well‐established feature of RA pathophysiology. As an essential component of SOD [37], copper contributes to antioxidant defence by facilitating free radical scavenging, thereby potentially mitigating oxidative stress [40]. Conversely, excessive copper can promote free radical generation via Fenton chemistry, exacerbating joint inflammation and tissue damage [41]. Clinical observations further reveal significantly elevated copper concentrations in the synovial fluid of RA patients compared to healthy controls [42], along with markedly increased levels of pro‐inflammatory cytokines such as TNF‐α and IL‐6 [43, 44].

However, copper deficiency also disrupts joint homeostasis. First, as an essential cofactor for LOX, copper insufficiency directly impairs LOX enzymatic activity, resulting in inadequate cross‐linking of the type II collagen network. This leads to a disorganized extracellular matrix architecture and compromised mechanical resilience, rendering cartilage susceptible to microdamage under load‐bearing conditions [14, 38, 45]. Second, copper deficiency weakens the antioxidant defence system by reducing the activity of copper/zinc superoxide dismutase (SOD). The consequent accumulation of superoxide anions causes oxidative injury to chondrocytes and matrix components, thereby accelerating cellular senescence and matrix degradation [40, 46]. Third, copper is essential for mitochondrial function, particularly as a prosthetic group for cytochrome c oxidase in the electron transport chain. Copper deficiency disrupts oxidative phosphorylation, leading to insufficient energy supply in chondrocytes and impairing their capacity for matrix synthesis and repair [47, 48]. Together, these deficits establish a vicious cycle in which a structurally vulnerable matrix, uncontrolled oxidative stress, and energy‐deprived chondrocytes converge to drive the initiation and progression of OA—echoing the progressive degeneration and cell death described at the outset.

#### 2.2.2 Cuproptosis in Osteoarthritis (OA)

Cuproptosis has emerged as a promising therapeutic target in OA. Clinical investigations have demonstrated significantly elevated serum copper levels in OA patients, reaching approximately 147.2 mcg/dL compared to healthy controls [49]. More importantly, increased copper concentrations have been specifically documented within the articular cartilage of OA patients [50], strongly implicating cuproptosis in OA pathogenesis, particularly in cartilage degeneration [51, 52].

The mechanistic link between cuproptosis and OA operates through multiple pathways. First, excessive copper ions activate the Fenton reaction, generating substantial ROS [23] that exacerbate oxidative stress, disrupt chondrocyte mitochondrial function, and promote the release of inflammatory cytokines including IL‐1β and TNF‐α, collectively accelerating cartilage breakdown [53, 54]. Second, intracellular copper accumulation directly induces cuproptosis, which interferes with LOX‐mediated collagen cross‐linking [55]. This impairment of extracellular matrix integrity accelerates chondrocyte death and matrix degradation [56], thereby driving OA progression.

In RA, a systemic autoimmune disorder characterized by persistent synovial inflammation and subsequent cartilage destruction, the pathological environment creates ideal conditions for cuproptosis initiation. This establishes a self‐perpetuating cycle where cuproptosis amplifies RA pathology, and RA inflammation further promotes copper‐mediated cell death [57]. The RA synovium exists in a hypoxic state [58], which stabilizes hypoxia‐inducible factor‐1α (HIF‐1α). HIF‐1α subsequently upregulates the copper transporter CTR1 [59], enhancing copper uptake by synovial cells and immune cells. Concurrently, inflammation itself elevates synovial fluid copper concentrations [60], establishing a high‐copper joint environment.

Furthermore, activated immune cells (e.g., T cells) and fibroblast‐like synoviocytes in RA exhibit enhanced aerobic glycolysis (the ‘Warburg effect’) [61], producing substantial lactic acid. Notably, both elevated copper and lactate represent key triggers of cuproptosis [2]. The resulting cuproptosis leads to massive cell rupture and release of pro‐inflammatory cytokines such as IL‐6, IL‐1β, IL‐8 and TNF‐α [62], intensifying the intra‐articular inflammatory cascade. While some studies suggest that TGF‐β [63] and IL‐10 [64] released during cuproptosis may exert immunomodulatory effects, the net outcome is sustained inflammation. This inflammatory milieu further worsens synovial hypoxia and lactate accumulation, creating a vicious cycle that continuously reinforces cuproptosis and disease progression.

### 2.3 Copper in Bone Tumours

#### 2.3.1 Copper Homeostasis in Osteosarcoma (OS)

OS represents the most common primary malignant bone tumour, with approximately two‐thirds of cases occurring in adolescents and young adults [65, 66]. This biologically complex malignancy typically arises in the metaphyseal regions of long bones, including the proximal tibia, distal femur, and proximal humerus [67]. Pulmonary metastasis develops frequently in OS patients, contributing to a poor prognosis with historically high mortality rates; the 5‐year survival for metastatic or recurrent disease remains alarmingly low at approximately 20%–30% [68]. Despite advances in multimodal therapy, significant clinical challenges persist, including chemotherapy resistance, disease recurrence and distant metastasis [69].

Notably, alterations in copper metabolism appear integral to OS pathogenesis. Elevated serum copper levels have been documented in canine OS models [70], with corresponding fluctuations in ceruloplasmin levels [71], suggesting a non‐incidental relationship between copper homeostasis and OS progression. Mechanistically, copper promotes OS development through multiple interconnected pathways:

First, copper plays a fundamental role in tumour angiogenesis. Early studies demonstrated that copper deficiency impairs corneal neovascularization in rabbits [72], while subsequent research established that solid tumours—including OS—produce abundant vascular endothelial growth factor (VEGF) [73], whose expression correlates with tumour burden [74]. Copper, via the transporter CTR1, enhances VEGF production and receptor activation, thereby stimulating endothelial cell proliferation and new vessel formation [75, 76]. This angiogenic switch is essential for supplying the rapid growth and invasive potential of both primary bone tumours and skeletal metastases [77, 78]. As a key factor in angiogenesis, VEGF facilitates the ‘blood supply’ of tumours, thereby promoting their growth [79, 80, 81].

Second, copper directly activates key oncogenic signalling pathways. It stimulates MEK1 and MEK2 kinases [82, 83], which serve as critical relays in the MAPK cascade. Following upstream RAF activation, MEK1/2 phosphorylate ERK1/2 with high specificity, transmitting proliferative signals that ultimately regulate nuclear gene expression and cell fate decisions [84, 85, 86]. As central components of the MAPK pathway [87], whose dysregulation is a cancer hallmark [88], MEK1/2 activation by copper significantly fuels tumourigenesis.

Third, Copper activates hypoxia‐inducible factor‐1α (HIF‐1α) [89]. The HIF family is inherently involved in the entire tumour development process and enhances the risk of tumour metastasis [90, 91]. As one of the most pivotal members of the HIF family [92], HIF‐1α upregulates a repertoire of oncogenes under prevalent hypoxic conditions [93], including those associated with angiogenesis (e.g., VEGF) [73], glycolysis (Warburg effect) [61], and cell invasion. Notably, HIF‐1α exerts a more pronounced effect in early‐stage clear cell renal cell carcinoma (ccRCC) [94] and neuroblastoma (NB) [95]. By regulating factors such as HIF‐1α, copper ions enable tumour cells to adapt to and proliferate in this harsh microenvironment, while also ‘educating’ surrounding immune cells to promote tumour progression.

Finally, copper enhances metastatic capability through extracellular matrix remodelling [96]. As an essential cofactor for LOX [14], copper enables collagen and elastin cross‐linking [97], generating a stiffened matrix scaffold conducive to tumour growth and local invasion. Additionally, LOX‐generated hydrogen peroxide activates Src/FAK signalling and epithelial‐mesenchymal transition (EMT) [98, 99, 100], critical processes for metastatic dissemination. The correlation between elevated LOX expression and increased invasiveness in breast cancer [101] underscores its importance in metastasis—a particular relevance for OS, which demonstrates pronounced pulmonary tropism.

Conversely, copper deficiency also contributes to the pathogenesis of bone tumours through multiple interconnected mechanisms. First, copper deficiency reduces LOX activity, leading to decreased collagen cross‐linking and compromised structural stability of the bone matrix. This alteration of the bone microenvironment may create permissive conditions for tumour cell invasion and growth [98, 102]. Second, as an integral component of copper/zinc superoxide dismutase (Cu/Zn‐SOD), copper deficiency impairs the body's capacity to scavenge ROS, thereby increasing oxidative stress levels. Elevated oxidative stress can induce DNA damage, gene mutations and chromosomal instability, all of which may promote tumorigenesis [37, 103]. Third, copper deficiency may also affect the immune microenvironment of bone tissue, reducing immune cell function and antitumor immune surveillance, thereby allowing abnormal cells to escape immune clearance more readily [104]. Collectively, copper deficiency may participate in the pathological processes associated with bone tumours by disrupting bone matrix architecture, enhancing oxidative stress, interfering with immune regulation, and altering cellular metabolism.

**FIGURE 3:**
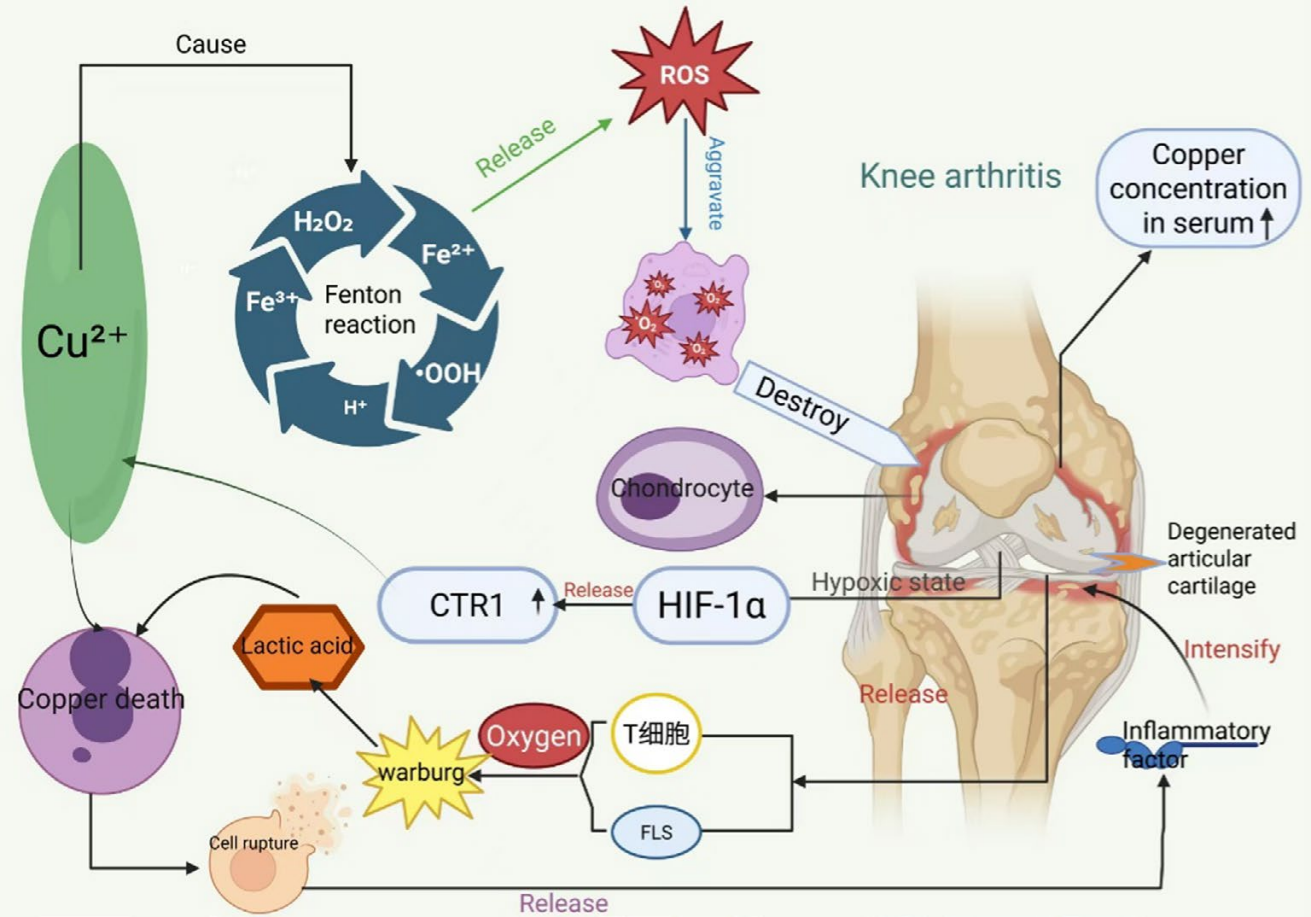
Mechanism diagram of copper's role in osteoarthritis: The pathological accumulation of copper ions can initiate the Fenton reaction, generating substantial amounts of reactive oxygen species (ROS) and inducing significant oxidative stress. This process promotes hypoxic damage and degeneration of articular cartilage in the knee joint, thereby driving the progression of degenerative knee osteoarthritis. Conversely, the hypoxic environment within the osteoarthritic joint cavity stimulates the expression of hypoxia‐inducible factor‐1α (HIF‐1α), which transcriptionally upregulates the copper transporter protein CTR1. This results in further copper accumulation in the joint tissues, ultimately triggering cuproptosis—a copper‐dependent form of regulated cell death. The ensuing massive cellular rupture releases intracellular contents that provoke a robust inflammatory response, significantly exacerbating arthritic pathology. Furthermore, in the copper‐enriched joint microenvironment, immune cells such as T lymphocytes and fibroblast‐like synoviocytes (FLS) exhibit enhanced aerobic glycolysis (the ‘Warburg effect’), leading to substantial lactate production. The coexistence of elevated lactic acid and high copper concentrations creates a synergistic effect that further potentiates the cuproptosis pathway, establishing a self‐reinforcing cycle that perpetuates joint destruction and disease chronicity.

In summary, copper contributes to bone tumour pathogenesis through four principal mechanisms: (1) supporting tumour vascularization via VEGF‐mediated angiogenesis; (2) driving proliferative signalling through direct MAPK pathway activation; (3) promoting microenvironment adaptation via HIF‐1α stabilization; and (4) facilitating metastatic dissemination through LOX‐dependent matrix remodelling and EMT induction. In contrast, the role of copper deficiency in bone tumours is primarily manifested through the following aspects: copper deficiency inhibits collagen cross‐linking and disrupts bone matrix structure; it impairs antioxidant defence and increases DNA damage; it affects the bone immune microenvironment and reduces immune surveillance capacity; and it interferes with mitochondrial metabolism and cellular energy metabolism (Figure 3).

#### 2.3.2 Cuproptosis Homeostasis in Osteosarcoma (OS)

Bone tumour development is a complex, multi‐step process [101]. Growing evidence indicates that dysregulated copper metabolism is closely associated with tumorigenesis. Copper supports cancer cell proliferation not only by fueling metabolic pathways but also through the release of bioactive molecules from dying cells—such as ATP and potassium ions [105]—which can act as signalling molecules or nutrient sources for residual tumour cells [106, 107, 108, 109]. Furthermore, copper accumulation in the local tumour microenvironment may trigger cuproptosis in stromal and normal cells, disrupting tissue homeostasis [110, 111] and creating a permissive niche for tumour expansion by eliminating competitors [102].

Cuproptosis also activates multiple oncogenic signalling cascades. Although copper ions can stabilize HIF‐1α under certain conditions, cuproptosis‐induced mitochondrial dysfunction—through disruption of the TCA cycle and oxidative phosphorylation—impairs vascular function and exacerbates hypoxia [112], which in turn strongly activates HIF‐1α [86, 89], further promoting tumour proliferation and metastasis.

A second major mechanism involves crosstalk between cuproptosis and the MAPK pathway, a central regulator of cell proliferation, survival and stress response [88]. Cuproptosis generates intense stress signals and robust mitochondrial and cytoplasmic ROS via copper‐driven Fenton reactions and mitochondrial collapse [23, 113]. ROS serve as key activators of the MAPK pathway, oxidatively modifying RAS to maintain it in a GTP‐bound, activated state [114, 115, 116, 117], thereby triggering MAPK signalling. Additionally, the inflammatory cytokines (e.g., TNF‐α, IL‐6 and IL‐17) and stress mediators released during cuproptosis [118] can transactivate growth factor receptors such as EGFR [119], leading to RAS activation via adaptor proteins like GRB2‐SOS and initiating the RAF–MEK–ERK cascade [120, 121], which further supports tumour survival and progression.

Finally, cuproptosis engages stress and inflammatory signalling networks to promote malignancy. Excess copper binds to XIAP, activating NF‐κB and enhancing invasive capacity [122, 123]. Concurrently, shedding of the Jagged1 extracellular domain modulates Notch signalling, facilitating metastatic dissemination to the lungs and other sites [124].

**FIGURE 4:**
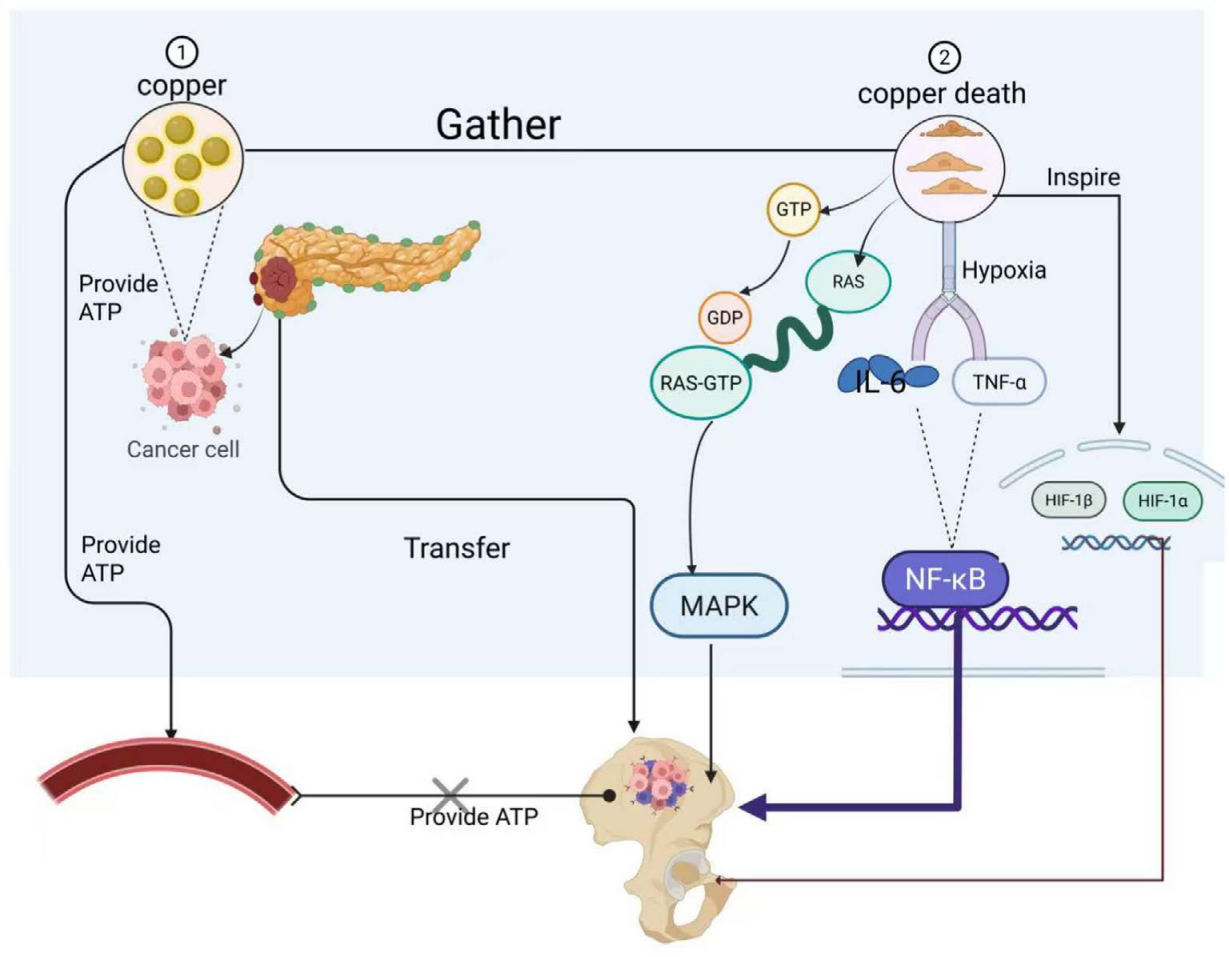
Mechanism diagram of copper and bone tumours. Under physiological conditions, copper ions contribute to cancer cell energy metabolism through their role in mitochondrial oxidative phosphorylation. Meanwhile, within the tumour microenvironment, copper potentiates the activity of vascular endothelial growth factor (VEGF)—a key angiogenic factor highly expressed in tumours—thereby indirectly supporting tumour proliferation by enhancing nutrient and oxygen supply. Consequently, disrupting the copper‐VEGF axis represents a promising therapeutic strategy for bone tumours. Pathologically, copper accumulation‐induced cuproptosis exacerbates intratumoral hypoxia, leading to stabilization and activation of the HIF‐1α/HIF‐1β signalling axis, which subsequently promotes cancer cell survival and proliferation. Furthermore, the inflammatory cascade triggered by cuproptosis results in release of cytokines such as TNF‐α and IL‐6, which activate NF‐κB and other pro‐survival pathways, thereby driving tumour cell expansion. Finally, cuproptosis‐mediated activation of the MAPK pathway further contributes to tumorigenesis. This occurs primarily through reactive oxygen species (ROS) generated during cuproptosis, which oxidatively modify and activate RAS proteins, maintaining them in a GTP‐bound state that perpetuates MAPK signalling and ultimately fosters bone tumour development.

In summary, copper and cuproptosis promote bone tumour progression through four interrelated mechanisms (Figure 4).

1. Cuproptosis eliminates normal stromal cells, freeing space and resources for tumour growth while providing nutritional support
2. It exacerbates hypoxia, activating the HIF‐1α pathway and driving cancer proliferation
3. It activates the MAPK pathway via ROS and transactivation mechanisms, enhancing proliferation, invasion and angiogenesis
4. It instigates an inflammatory tumour‐promoting microenvironment through cytokine release and NF‐κB/Notch activation, further supporting immune evasion and metastatic spread

## 3 Cuproptosis and Other Forms of Cell Death

### 3.1 Differences and Connections Between Cuproptosis, Ferroptosis, Apoptosis and Autophagy

Beyond the role of copper in disease through mitochondria‐induced cuproptosis, accumulating evidence suggests that multiple cell death pathways are also engaged within complex biological systems. Cuproptosis, ferroptosis, apoptosis and autophagy are all critical modes of cell death or fate regulation. However, they exhibit fundamental differences in terms of inducing factors, molecular mechanisms, organelle dependency and biological functions while also displaying extensive crosstalk within signalling networks.

Ferroptosis is a form of regulated cell death that has garnered extensive attention in recent years, characterized by uncontrolled, iron‐dependent lipid peroxidation [125]. This process primarily results from glutathione (GSH) depletion or inactivation of glutathione peroxidase 4 (GPX4), leading to the peroxidation of polyunsaturated fatty acid‐containing phospholipids (PUFA‐PLs) and ultimately disrupting cell membrane integrity [126]. Iron ions play a critical role in this process by promoting the generation of ROS via the Fenton reaction [41]. Morphologically, ferroptosis is characterized by shrunken mitochondria with increased membrane density, while the nuclear structure remains relatively intact [127].

Autophagy is a highly conserved lysosome‐dependent degradation process that encapsulates damaged organelles or proteins within double‐membrane structures termed autophagosomes, which subsequently fuse with lysosomes to achieve degradation. Autophagy typically functions as an adaptive response to nutrient deprivation or stress stimuli, thereby maintaining cellular homeostasis. However, under conditions of sustained or excessive activation, autophagy can be converted into a form of cell death [128, 129]. Selective autophagy, such as ferritinophagy, contributes to the induction of ferroptosis by regulating iron metabolism [130].

Apoptosis is the most classic form of programmed cell death, which is tightly regulated by genes and primarily relies on the cascade activation of caspase family proteins [131]. Its activation pathways include the intrinsic (mitochondrial) pathway and the extrinsic (death receptor) pathway. In the intrinsic pathway, increased permeability of the outer mitochondrial membrane leads to the release of cytochrome c and subsequent activation of caspase‐9, whereas the extrinsic pathway involves caspase‐8 activation mediated by receptors such as Fas/FasL [132]. Apoptotic cells exhibit characteristic features including cell shrinkage, chromatin condensation, and formation of apoptotic bodies, and typically do not elicit an inflammatory response.

In summary, ferroptosis is centred on lipid peroxidation, autophagy is characterized by lysosomal degradation, and apoptosis relies on caspase‐mediated programmed regulation, with their specific differences summarized in Table 1. Together, these three processes constitute a fundamental basis for the regulation of cell fate.

**TABLE 1:** Comparison of mechanistic differences among four cell death modalities.

	Cuproptosis	Ferroptosis	Apoptosis	Autophagy
Inducing factors	Copper ion overload (Cu^+^)	Iron‐dependent lipid peroxidation	DNA damage, oxidative stress, cytokines, etc.	Nutrient deficiency, stress, injury
Core mechanism	Copper‐binding lipoylated proteins → protein aggregation → loss of Fe–S proteins → proteotoxic stress	Fe^2+^‐catalysed lipid peroxidation (Fenton reaction)	Caspase cascade activation	Autophagosome formation and degradation of cellular components
Key molecules	FDX1, LIAS, DLAT, lipoylated proteins	GPX4, Fe	The caspase family of proteases	ATG proteins
Metabolic dependency	TCA	Lipid metabolism, iron metabolism	Low energy dependence	Energy‐dependent process
Nuclear changes	Intact, no chromatin condensation	Intact, no chromatin condensation	Chromatin condensation, margination, DNA laddering	Intact
Major signalling pathways	Copper homeostasis regulation, mitochondrial metabolism	Iron metabolism, lipid metabolism, oxidative stress	p53, Bcl‐2 family, death receptor pathway	mTOR, AMPK signalling pathways
Typical inhibitors	Copper‐chelating agent (TTM)	Ferrostatin‐1 [133], deferoxamine, liproxstatin‐1 [134]	Pyroptosis with pan‐caspase inhibitor [135]	Caspase [136]

Cuproptosis, ferroptosis, autophagy, and apoptosis constitute a tightly intertwined regulatory network within cells. They share upstream signals such as oxidative stress, mitochondrial dysfunction, and metal ion metabolic disturbances and mutually influence one another through key molecular hubs. Among them, the connection between cuproptosis and ferroptosis is particularly close. The loss of Fe‐S cluster proteins during cuproptosis can lead to iron metabolic disorders and indirectly promote ferroptosis. Moreover, copper ions can inhibit the activity of GPX4, a core inhibitory factor of ferroptosis, resulting in a synergistic amplification effect between the two under pathological conditions [137]. Autophagy exhibits bidirectional regulatory relationships with these two death modalities: on one hand, autophagy can inhibit cuproptosis and ferroptosis by clearing damaged mitochondria and toxic proteins; on the other hand, selective autophagy forms such as ferritinophagy release free iron and thereby promote ferroptosis [138]. Additionally, when autophagic flux is blocked or excessively activated, autophagy may shift towards a pro‐death effect. Although cuproptosis and apoptosis are independent of each other in terms of their core execution mechanisms (cuproptosis is caspase‐independent), the severe mitochondrial damage induced by cuproptosis can release cytochrome c, thereby triggering downstream apoptotic pathways [83], making apoptosis often serve as a downstream amplification link of cuproptosis. The interplay between autophagy and apoptosis is the most complex: autophagy generally bidirectionally regulates apoptotic sensitivity by clearing damaged mitochondria and degrading anti‐apoptotic proteins, whereas during the execution phase of apoptosis, caspases can cleave key autophagic proteins and inactivate autophagy [139]. Collectively, these four modes of cell death do not occur in isolation but form a complex crosstalk network through metal metabolism, mitochondrial function, redox balance and core signalling pathways. Within pathophysiological contexts such as the bone cell microenvironment, they often exhibit dynamic relationships of coexistence, synergy, or antagonism.

### 3.2 Interactions of Cuproptosis, Ferroptosis, Apoptosis and Autophagy in the Bone Microenvironment

Within the bone cell microenvironment, cuproptosis and ferroptosis are most closely interconnected due to their shared involvement in metal metabolic disturbances and oxidative stress pathways. During copper overload‐induced cuproptosis, the loss of mitochondrial Fe–S cluster proteins disrupts intracellular iron homeostasis, leading to an increase in free iron ions, which subsequently catalyse lipid peroxidation via the Fenton reaction and indirectly enhance cellular sensitivity to ferroptosis [140]. Concurrently, copper ions can directly inhibit the activity of GPX4, a core suppressor of ferroptosis, resulting in a synergistic amplification of these two death modalities under pathological conditions such as OP and OA. In these settings, cuproptosis in osteoblasts and ferroptosis in osteoclasts occur simultaneously, producing a dual hit of reduced bone formation and increased bone resorption, thereby accelerating bone loss [141, 142].

Autophagy acts as a key regulatory switch for cuproptosis and ferroptosis within the bone microenvironment. On one hand, autophagy effectively inhibits the occurrence of cuproptosis and ferroptosis by selectively clearing damaged mitochondria (mitophagy) and aggregated toxic proteins, thereby maintaining the survival of osteoblasts and chondrocytes [143]. On the other hand, selective autophagy forms such as ferritinophagy degrade ferritin and release free iron, which in turn promotes ferroptosis [144]. In OA, autophagic activity in chondrocytes declines with age, leading to a marked increase in their susceptibility to both ferroptosis and cuproptosis [145, 146]. In the context of OS therapy, autophagy inhibitors have been shown to enhance the cuproptotic effect induced by copper ionophores, suggesting potential for combination therapeutic strategies. Thus, the dual role of autophagy positions it as a central node in the crosstalk among multiple cell death pathways within the bone microenvironment.

Although apoptosis is independent of cuproptosis and ferroptosis in terms of its core execution mechanism (caspase‐independent), it often serves as a downstream amplification link for these death modalities within the bone microenvironment. Severe mitochondrial damage induced by cuproptosis and ferroptosis leads to collapse of the mitochondrial membrane potential and release of cytochrome c, which subsequently activates caspase‐dependent apoptotic pathways, thereby establishing a cascade linking apoptosis with cuproptosis and ferroptosis [147]. In arthritis, particularly OA, inflammatory cytokines and mechanical stress jointly elevate ROS levels, promoting both ferroptosis and apoptosis in chondrocytes, while copper homeostasis disruption may further exacerbate mitochondrial damage and participate in cuproptosis [146]. In bone tumours, cancer cells often evade apoptosis and ferroptosis by enhancing antioxidant capacity or modulating autophagy; consequently, inducing cuproptosis has emerged as a potential therapeutic strategy that may synergize with other death modalities to enhance anti‐tumour efficacy [148]. In OP, decreased osteoblast function coexists with enhanced osteoclast activity, and oxidative stress‐driven ferroptosis and apoptosis collectively contribute to reduced bone formation, while copper metabolic abnormalities may further aggravate this process by impairing mitochondrial function.

In summary, across distinct pathological conditions such as arthritis, bone tumours, and OP, cuproptosis, ferroptosis, apoptosis and autophagy do not occur in isolation. Instead, they form a regulatory network of mutual promotion or inhibition through oxidative stress, mitochondrial function and metal ion metabolic pathways. This synergistic or antagonistic interplay among multiple cell death modalities collectively determines the fate of bone cells and the progression of disease. These insights also suggest that targeting a single cell death pathway may be of limited efficacy in the treatment of orthopaedic diseases, whereas strategies based on the combined regulation of multiple pathways hold greater potential value.

## 4 Treatment for Osteoporosis, Osteoarthritis and Bone Tumours

See Figure 5.

**FIGURE 5:**
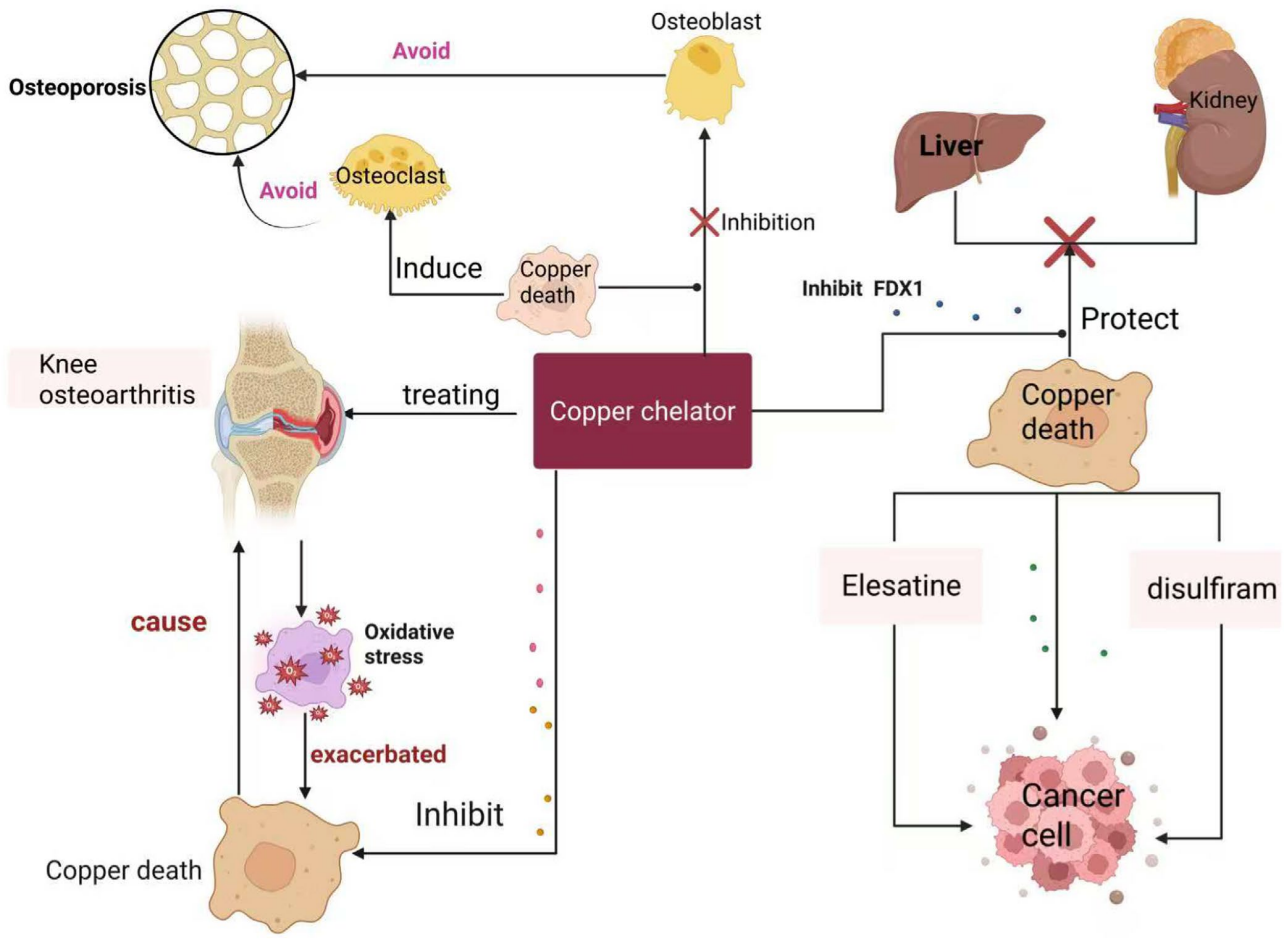
Mechanism diagram of regulating copper (Cu^2+^) for the treatment of osteoporosis, osteoarthritis, and bone tumours via copper chelators. Copper chelators can suppress cuproptosis (copper‐induced cell death), thereby preventing osteoblasts from dying due to intracellular copper overload. Conversely, cuproptosis induces apoptosis in osteoclasts, which in turn helps to prevent osteoporosis. Secondly, cuproptosis can exacerbate knee osteoarthritis, while knee osteoarthritis, in turn, intensifies cuproptosis by elevating oxidative stress reactions through internal inflammatory factors. Therefore, copper chelators can not only inhibit cuproptosis but also directly treat knee osteoarthritis. Copper chelators can protect liver and kidney function by inhibiting cuproptosis, preventing damage to these organs. However, cuproptosis can be induced within tumour cells when copper is delivered via disulfiram and elesclomol carriers, leading to copper overload in tumour cells. This overload triggers cuproptosis within the tumour cells, ultimately resulting in their apoptosis.

### 4.1 Regulating Cuproptosis for the Treatment of Osteoporosis (OP)

OP can arise from multiple etiological factors, one of which is the disruption of cellular homeostasis‐induced OP. Copper is an essential micronutrient for maintaining cellular homeostasis; thus, the occurrence of cuproptosis (copper‐induced cell death) within cells can trigger metabolic disorders, thereby exacerbating the severity of OP [149]. However, to develop OP treatments targeting cuproptosis regulation, a comprehensive understanding of the pathological crosstalk between OP and cuproptosis is prerequisite. This association primarily stems from the observation that cuproptosis in osteoblasts impairs their bone‐forming capacity, leading to bone mass loss and subsequent development of OP. Therefore, the therapeutic strategies for OP via cuproptosis inhibition mainly involve the following aspects.

Firstly, the most direct approach is the application of copper chelators. Copper chelators, which can bind to copper ions, have been utilized in the treatment of various diseases [150], including orthopaedic disorders, cancer, and cardiovascular diseases. For example, drugs such as aminopropionic acid, zinc sulphate (ZS), and D‐penicillamine have been employed for these therapeutic purposes [151]. These agents act as ‘magnets’ to scavenge excessive and toxic free copper ions in the body. A critical step in this strategy is bone‐targeted delivery [150]; specifically, through the use of nanotechnology or conjugation of drugs with bisphosphonates (a bone‐affinity molecule), therapeutic agents can be precisely enriched on the bone surface, internalized by osteoblasts, and subsequently regulate cuproptosis and copper metabolism while minimizing systemic side effects.

Secondly, the development of cuproptosis inhibitors—particularly small‐molecule inhibitors targeting key cuproptosis‐related proteins (e.g., FDX1) [26]—can directly block cellular death signalling pathways. When combined with conventional therapies, such as the co‐administration of bisphosphonates and bone anabolic agents (e.g., teriparatide) [152, 153], a synergistic effect of ‘protecting osteoblasts against cuproptosis + stimulating osteoblast activity’ can be achieved, leading to improved therapeutic outcomes.

Finally, for more in‐depth investigations, a dual‐pronged strategy should be explored to harness cuproptosis for OP treatment: protecting osteoblasts while simultaneously inhibiting osteoclasts, thereby preventing the initiation or progression of OP. Hong et al. [154] developed a novel MgO‐1Cu nanocomposite by combining MgO nanoparticles with a CuSO_4_ solution at a specific ratio. This material enhanced osteoblast proliferation and differentiation, suppressed osteoclast formation, and exhibited effective antibacterial activity; however, a notable limitation was the inherent toxicity of the composite.

Notably, copper homeostasis plays a pivotal role in bone health. Copper deficiency can lead to increased bone mineral density (often accompanied by impaired bone quality) and enhanced bone fragility [155]. Furthermore, murine studies [27, 156] demonstrated that induced copper deficiency resulted in significant reductions in bone mineral density (BMD) in the fifth lumbar vertebra and proximal femur, as confirmed by quantitative analyses. Therefore, maintaining copper homeostasis and achieving efficient, targeted delivery of copper ions or copper ion carriers to osteoclasts at skeletal sites—while avoiding off‐target effects on other organs (e.g., liver and brain)—critically depends on advancements in nanotechnology and biomaterials.

Nevertheless, current research in this field remains predominantly limited to in vitro cellular and in vivo animal experimental stages, with a substantial gap to be addressed before transitioning to human clinical trials and subsequent market approval. Moreover, for the treatment of cuproptosis‐induced OP, further investigations are urgently required to translate these preclinical findings into clinical applications.

The overall therapeutic mechanisms of copper regulation in orthopaedic diseases are summarized in Figure 5.

### 4.2 Modulating Cuproptosis for the Treatment of Osteoarthritis (OA)

The connection between cuproptosis and osteoarticular diseases is particularly evident in OA, where chondrocyte cuproptosis contributes to cartilage matrix degradation, progressive joint degeneration, and subsequent synovial inflammation [157]. Clinical evidence consistently shows elevated serum copper levels in OA patients compared to healthy controls [158], with similarly increased copper concentrations observed in synovial fluid [159], establishing a clear association between copper dysregulation and OA pathogenesis [160].

Several therapeutic approaches targeting copper metabolism have shown promise. Tetrathiomolybdate (TM), an approved anti‐copper agent for Wilson's disease [161, 162], demonstrates anti‐arthritic properties by reducing intracellular copper levels and suppressing joint inflammation and destruction. From a genetic perspective, Mendelian randomization analysis suggests copper complexes may selectively clear senescent and necrotic cells while promoting chondrocyte regeneration [163]. Additionally, advanced cartilage tissue engineering utilizes nanomaterials—including thermoresponsive hydrogels, alginate and gelatin‐based biopolymers—to enhance chondrocyte function and potentially prevent OA development [164].

For patients experiencing acute OA flares, where pain management becomes paramount, breaking the cycle of oxidative stress and pro‐inflammatory cytokines is crucial. The hybrid thermosensitive hydrogel (HPP@Cu gel), incorporating copper nanoparticles with bioactive components, has emerged as a promising intervention. This innovative material scavenges reactive oxygen and nitrogen species (RONS) at inflammatory sites, promotes M2 macrophage polarization, suppresses M1 macrophage activation, and inhibits pro‐inflammatory cytokine production. In OA rat models, HPP@Cu gel significantly attenuated cartilage degeneration while controlling inflammatory cytokine proliferation.

RA, characterized by its autoimmune aetiology [165], can lead to severe systemic complications affecting respiratory, cardiovascular, and other vital systems in advanced stages [166, 167]. Its pathogenesis involves autoimmune‐mediated synovitis, pannus formation, and progressive destruction of articular cartilage and subchondral bone, ultimately causing joint deformity and functional loss [168]. Research has identified 53 copper toxicity‐related genes associated with RA pathogenesis, including miRNAs regulating PDHB, FDX1‐mediated fatty acid oxidation and HIF‐1/CDKN2A‐governed cellular senescence pathways [169, 170].

Two primary strategies have emerged for targeting cuproptosis in RA management. The first employs copper chelators, such as the established D‐penicillamine complex [171, 172], which modulates copper‐mediated redox reactions to achieve clinical benefit. The second, the strategic induction of cuproptosis represents a promising therapeutic approach for RA. In principle, this strategy aims to selectively trigger cuproptosis in pro‐inflammatory cells—primarily synovial fibroblasts and activated immune cells [173]—while minimizing damage to healthy cells. This can be achieved by employing copper ionophores (e.g., targeting PDHA1 and DLST [174]) to transport extracellular copper into mitochondria, leading to copper overload within inflammatory cells and subsequent induction of cuproptosis [175].

Despite compelling evidence linking cuproptosis to OA pathophysiology, significant translational challenges must be overcome before cuproptosis can be effectively targeted for OA therapy.

### 4.3 Modulating Cuproptosis as a Therapeutic Strategy for Bone Tumours

Substantial clinical evidence indicates that cancer patients frequently exhibit dysregulated copper protein levels [176]. Cuproptosis contributes to malignant progression through three principal mechanisms [177, 178]: induction of angiogenesis, activation of pro‐tumour signalling pathways, and remodelling of the tumour microenvironment (TME). Consequently, targeting cuproptosis has emerged as a promising therapeutic strategy for bone cancers. Copper accumulation functions as a ‘collaborator’ in tumour progression through multiple mechanisms: elevated copper levels within the TME stimulate angiogenesis [106, 179], thereby enhancing nutrient and oxygen delivery to support rapid tumour growth; aberrant copper activates key oncogenic pathways including MAPK/ERK [82, 83] and PI3K/AKT [166, 180], enabling bone tumour cells to evade apoptosis while enhancing their migratory and invasive capabilities; and copper remodelling immune cell function establishes an immunosuppressive TME that facilitates tumour immune evasion [181, 182].

Therapeutic approaches targeting copper metabolism in bone tumours exploit this metal's dual nature. To reduce pro‐tumour copper levels, chelators including D‐penicillamine [183], TM [184] and trientine [185] can deplete intratumoral copper pools. Conversely, the elevated copper requirements of many cancer cells, including OS, create an opportunity for selectively inducing cuproptosis. Copper ionophores such as elesclomol [2] can transport exogenous copper into malignant cells, generating artificial ‘copper overload’ that triggers selective cuproptosis [186] with minimal impact on normal cells.

This approach holds particular promise for overcoming therapy resistance. Bone tumours frequently develop resistance to conventional agents like cisplatin [187] and doxorubicin [188, 189]. Since cuproptosis operates through mechanisms distinct from apoptosis [2], it may effectively eliminate apoptosis‐resistant tumour cells, offering a novel strategy to circumvent drug resistance [190]. Furthermore, combining cuproptosis inducers with conventional chemotherapy, radiotherapy, or immunotherapy may yield synergistic benefits. Recent pan‐cancer analyses reveal significant correlations between cuproptosis and immune cell infiltration patterns [191], suggesting potential for combined immunotherapeutic approaches. The cuproptosis‐related gene FDX1 demonstrates particular promise as both a prognostic biomarker and therapeutic target [192], with cuproptosis‐related gene sets showing extensive cross‐talk across multiple solid tumours [193]. Pan‐cancer analysis of these genes may reveal novel strategies for targeting cuproptosis in OS.

Table 2 summarizes the clinically used drugs that induce or inhibit cuproptosis.

**TABLE 2:** Clinically used drugs that induce or inhibit cuproptosis.

Classification	Drug	Mechanism
Induce cuproptosis	Elesclomol	Elesclomol enters cells alongside copper ions via specific membrane transporters. Once intracellular, the Elesclomol‐Cu^2+^ complex elevates mitochondrial free copper levels in a ROS‐dependent manner [194]. This copper accumulation induces aggregation of lipoylated TCA cycle enzymes and functional impairment of iron–sulphur cluster proteins [195], culminating in cell death through cuproptosis [196]
	Disulfiram (DSF)	Disulfiram, originally developed as an alcohol‐aversion therapeutic [197], functions as an effective copper ionophore upon intracellular binding with copper ions. The resulting Disulfiram‐Cu^2+^ complex accumulates within cells and induces substantial oxidative stress, ultimately triggering cell death. For example, the Disulfiram/Cu^2+^ combination has been demonstrated to induce apoptotic cell death in hepatocellular carcinoma cells [198] and to activate ULK1‐mediated autophagy, thereby suppressing proliferation in colorectal cancer models [199]. These mechanisms establish Disulfiram as a potent inducer of cuproptosis
	Trientine	Trientine is approved for treating Wilson's disease in patients intolerant to penicillamine [200]. Although historically classified as a copper chelator [201] and explored for potential cardioprotective applications, its principal use remains the management of Wilson's disease. Emerging evidence indicates that trientine exerts dual regulatory effects on copper homeostasis: it promotes urinary copper excretion to alleviate copper overload in type 2 diabetes mellitus (T2DM) [202], while also ameliorating copper deficiency in ischemic heart disease (IHD) [203]. By sequestering and eliminating systemic copper, trientine removes the fundamental trigger for cuproptosis, thereby preventing activation of this cell death pathway. This copper‐depleting mechanism underpins its efficacy as a first‐line treatment for Wilson's disease
	Clioquinol (CQ)	Clioquinol (CQ) was historically employed in the treatment of diarrheal disorders and cutaneous infections [204, 205]. Recent investigations, however, have demonstrated its capacity to form complexes with copper ions (Cu^2+^) that are efficiently internalized by cells, resulting in significant intracellular copper overload [206]. This excessive copper accumulation disrupts mitochondrial function by promoting abnormal aggregation and functional impairment of proteins involved in cellular energy metabolism [207], ultimately leading to metabolic collapse and apoptotic death in cancer cells [46, 208]
Inhibit cuproptosis	Tetrathiomolybdate (TTM)	Upon entering the systemic circulation, tetrathiomolybdate (TTM) enhances glutathione (GSH) synthesis, facilitating endogenous copper chelation and bolstering chondrocyte resilience [209]. This process depletes bioactive free copper pools within cells. Simultaneously, in the intestinal lumen, TTM selectively binds dietary copper ions (Cu^2+^) to form insoluble complexes that are excreted in faeces [210], thereby inhibiting copper absorption at its primary site of entry. Through this dual mechanism—combining intracellular chelation and luminal sequestration—TTM effectively reduces systemic and intracellular copper availability, preventing the initiation of cuproptosis at its source
	D‐penicillamine (DPA)	D‐penicillamine demonstrates high binding affinity for Cu^2+^ ions in vivo, forming stable, soluble complexes that undergo hepatorenal and enteric processing, with excess copper ultimately excreted predominantly in faeces. This represents the most direct pathway for copper depletion. By this mechanism, D‐penicillamine substantially reduces free copper concentrations in both intracellular and extracellular compartments—particularly within mitochondria—independently of tricarboxylic acid (TCA) cycle involvement [211, 212]. This copper‐lowering effect enhances cellular resistance to cuproptosis by alleviating copper‐mediated mitochondrial dysfunction and oxidative stress
	Tetracycline	Tetracycline, widely used in managing intestinal disorders [213], also functions as an effective chelating agent. Research has demonstrated that tetracycline forms a stable Cu‐tetracycline complex through Cu^2+^ chelation, effectively acting as a molecular sponge that sequesters and immobilizes intracellular copper ions, thereby rendering them biologically inactive [214, 215]. In essence, by depleting biologically available copper pools, tetracycline effectively inhibits the initiation of cuproptosis

Additionally, radiotherapy enhances intracellular ROS levels, which synergize with copper ions to potentiate cuproptosis [216]. This combination approach may enable selective tumour cell killing while preserving normal tissue, representing a promising direction for future therapeutic development.

### 4.4 Clinical Agents for Inducing or Inhibiting Cuproptosis

See Table 2.

## 5 Summary and Outlook

In recent years, research on copper biology has expanded remarkably. As an essential trace element, copper ions (Cu^2+^) play a critical role in maintaining physiological homeostasis through their involvement in key biochemical processes. Under normal conditions, copper supports vital functions such as antioxidant defence and angiogenesis. However, in a pathological context, abnormal copper accumulation triggers cuproptosis—a double‐edged sword that exerts contrasting effects depending on the cellular environment. While it can selectively eliminate cancer cells, copper overload may also induce death in normal immune cells, highlighting its potential as a novel target for oncological therapy.

Although the physiological importance of copper is well established, the precise mechanisms of cuproptosis and its translational applicability require further elucidation. This review has systematically examined the role of copper in cellular processes and explored the therapeutic potential of modulating copper homeostasis in orthopaedic diseases, including OA, RA, OP and OS. Nevertheless, translating these insights into clinical practice faces considerable challenges. For instance, disease‐specific optimal copper thresholds remain undefined, particularly for OP and OS, where the precise copper concentrations required for maximal efficacy are still unclear.

Additional hurdles include the targeted delivery of copper ionophores to bone tumour sites while sparing healthy tissues such as the liver and brain. Furthermore, cell‐type‐specific gene editing models could help decipher the interplay between cuproptosis and other cell death pathways, such as ferroptosis and apoptosis. At the therapeutic level, future studies should prioritize the development of synergistic strategies—for example, combining cuproptosis inducers with immunotherapy for bone malignancies or designing multifunctional agents to disrupt the oxidative stress–cuproptosis cycle in arthritic conditions.

Through interdisciplinary collaboration, the precise modulation of cuproptosis holds promise for revolutionizing the diagnosis and treatment of orthopaedic disorders. Current research lays a critical foundation for future exploration, and as our understanding of cuproptosis in disease deepens, subsequent investigations are expected to yield substantial advances in both conceptual and clinical realms.

## Author Contributions

**Chaoshuai Wang:** writing – original draft, conceptualization, visualization. **Pengfei An:** conceptualization, methodology.

## Funding

The authors have nothing to report.

## Conflicts of Interest

The authors declare no conflicts of interest.

## Data Availability

The authors have nothing to report.
